# Largazole targets Musashi protein expression via miR-125b-5p and sensitizes triple-negative breast cancer cells to radiation

**DOI:** 10.3389/fphar.2026.1745079

**Published:** 2026-06-23

**Authors:** Kathrin A. Gurke Brücksken, Lea Lappe, Qi-Yin Chen, Hendrik Luesch, Eberhard Korsching, Lasse Reichstein, Anne Marzi, Nancy A. Espinoza-Sánchez, Timo Habig, Ezequiel M. Fuentes-Pananá, Björn Kemper, Joke Tio, Martin Götte, Hans Theodor Eich, Burkhard Greve, Fabian M. Troschel

**Affiliations:** 1 Department of Radiation Oncology, University Hospital Münster, Münster, Germany; 2 Department of Medicinal Chemistry and Center for Natural Products, Drug Discovery and Development, University of Florida, Gainesville, FL, United States; 3 Program in Cancer and Stem Cell Biology, Duke-NUS Medical School, Singapore, Singapore; 4 Cancer and Complex Systems Research Group, Medical Faculty, University of Münster, Münster, Germany; 5 Biomedical Technology Center, Medical Faculty, University of Münster, Münster, Germany; 6 Department of Gynecology and Obstetrics, University Hospital Münster, Münster, Germany; 7 Research Unit on Virology and Cancer, Children’s Hospital of Mexico ‘Federico Gómez’, Mexico City, Mexico

**Keywords:** cancer stem cells, DNA repair, HDAC, miR-125b-5p, Musashi RNA-binding proteins, radiosensitivity, triple-negative breast cancer

## Abstract

**Background:**

Recent findings implicate the histone deacetylase (HDAC) inhibitor largazole as an inhibitor of Musashi RNA-binding protein function. Here, we assess this interplay and evaluate the relevance of largazole for triple-negative breast cancer (TNBC) progression and resistance to radiotherapy.

**Methods:**

Primary patient-derived TNBC cells and cell lines were treated with largazole and cell vitality, proliferation, motility, cell cycle, DNA synthesis as well as repair, and stemness were analyzed via MTT assay, digital holographic microscopy, and flow cytometry. To unravel the connection between largazole treatment and Musashi expression, miR-125b-5p was assessed after largazole treatment, and overexpressed as well as downregulated in TNBC wildtype cells to determine influence on Musashi levels. Targeted mRNA and protein expression analyses were complemented with RNA sequencing data after largazole treatment. Finally, DNA double strand breaks and post-radiogenic survival were quantified using γ-H2AX, 53BP1, and clonogenic assays.

**Results:**

Largazole showed reduced metabolic activity in TNBC, but not in non-malignant cultures. Largazole treatment strongly abrogated proliferation, DNA synthesis, cell motility, and induced a cell cycle arrest. Levels of the Musashi proteins were downregulated after largazole treatment via upregulation of the miR-125b-5p. Protein expression and RNA sequencing analysis indicated a loss of cancer stemness-, cell cycle progression-, and DNA repair-associated signaling. Consequently, radiotherapy-induced DNA double strand breaks were increased while post-radiogenic cell survival was decreased in largazole-treated samples.

**Conclusion:**

The HDAC inhibitor largazole compromises tumor growth and motility and downregulates the Musashi proteins via the miR-125b-5p in TNBC. Additionally, largazole acts as a radiosensitizer by attenuating DNA repair, therefore supporting therapeutic efficacy.

## 1 Introduction

Breast cancer remains the most common malignancy in females with a yearly incidence of 2.3 million new cases worldwide. Recent studies indicate that by 2050 the breast cancer incidence will increase by 38% while the mortality will rise by 68%, suggesting a growing need to improve therapy efficacy (Kim et al., 2025). 15%–20% of cases are classified as triple-negative breast cancer (TNBC) (Abuhadra et al., 2022). TNBC is a molecular subtype which is difficult to treat, as it lacks targetable receptors (i.e., estrogen receptor, progesterone receptor and human epidermal growth factor receptor 2) (Gluz et al., 2009; Kumar et al., 2023), limiting treatment options (Xiong et al., 2024). Current treatment strategies consist of a combination of chemotherapy (platinum and taxane), immunotherapy, surgery, and irradiation, depending on spread of disease (Kim et al., 2024; Schmid et al., 2024). Despite this multimodal approach, the 5-year survival rate for women with TNBC is 86.6%, compared with 93% for other breast cancer subtypes (Schmid et al., 2024; Bauer et al., 2007). Due to the limited systemic treatment options beyond chemotherapy and immunotherapy and the aggressive tumor histology, metastatic TNBC has a median overall survival of 13 months (Kesireddy et al., 2024) which is comparable only to highly malignant poor-prognosis tumors such as glioblastoma (Troschel et al., 2023).

Consequently, novel therapeutic options are investigated to improve cancer therapy, including small molecule inhibitors. In this setting, histone deacetylases (HDACs) have been shown to be overexpressed in multiple malignancies (Li and Seto, 2016) including HDAC3 and HDAC8 in TNBC (An et al., 2020; Cui et al., 2018) HDACs are known to influence epigenetic changes, including loss of acetylation (Al-Awadhi et al., 2020), subsequently governing cellular gene expression. Xu and colleagues demonstrated that HDAC2 expression was significantly increased in TNBC compared with other breast cancer subtypes. Furthermore, HDAC2 knockdown has been shown to suppress tumor-promoting mechanisms in TNBC (Xu et al., 2021). Additionally, HDAC3 overexpression has been correlated with poor overall survival in invasive ductal breast cancer (Cui et al., 2018). Several HDAC inhibitors such as SAHA, PXD101, LBH589 and FK228, have been approved by the FDA for use in cancer therapy (Al-Awadhi et al., 2020). In particular, class I HDACs (including HDAC 1, 2, 3, and 8) which focus on transcriptional control, have been identified as therapeutic targets. Largazole, a potent class I selective histone deacetylase inhibitor (Al-Awadhi et al., 2020), was discovered by Luesch and colleagues from a marine cyanobacterium (Hong and Luesch, 2012). Largazole treatment demonstrated anti-proliferative effects in different cancer cell lines (Qiu et al., 2021) and anti-invasive properties in TNBC (Law et al., 2013).

Based on molecular docking analysis Wang et al. suggested that largazole binds to the RNA-binding protein Musashi-2. However, largazole is a prodrug and inevitably undergoes protein-assisted hydrolysis in human cells or cell culture, resulting in a largazole thiol and making the docking model unlikely to be accurate (Hong and Luesch, 2012; Liu et al., 2010). Nonetheless, Wang *et al.* convincingly demonstrated a downregulation of Musashi-2 after largazole application, suggesting an unidentified functional relationship (Wang et al., 2020).

The Musashi RNA-binding proteins were first described in 1994 in the model organism *Drosophila* by Nakamura et al. (1994). There are two related genes, Musashi-1 (MSI1) and Musashi-2 (MSI2), which share approximately 90% homology in their RRM2 domain and have similar RNA-binding specificities (Okano et al., 2005; Sakakibara et al., 2001). Both are involved in the regulation of post-transcriptional gene expression, contributing to cancer initiation, progression, cancer stemness (Götte et al., 2008; Fox et al., 2015; Falke et al., 2022; Habig et al., 2025), and metastasis (Troschel et al., 2021). Additionally, both MSI proteins are overexpressed in breast cancer (Sicking et al., 2023), where high MSI1 levels are considered prognostically unfavorable (Wang et al., 2010). Previous studies by our group showed that modulating MSI protein expression, either through knockdown approaches or inhibitor treatment, may sensitize cells to radiation (Brücksken et al., 2025; Haiduk et al., 2023).

In this study, we aim to analyze the impact of largazole in TNBC as a potential therapeutic agent, as well as its effect on the regulation of the MSI proteins.

## 2 Materials and methods

### 2.1 Cell culture

The triple-negative breast cancer cell lines SUM149PT (authenticated in 2022), MDA-MB-231 (obtained from DSMZ, Braunschweig, Germany in 2023), MDA-MB-468 (obtained from DSMZ, Braunschweig, Germany, in 2023), and the primary human breast cancer cell culture UIVC-IDC-4 (Espinoza-Sánchez et al., 2017) were used for the *in vitro* experiments. The SUM149PT cells were cultured in Ham’s F12 Media (PAN-Biotech, Cat. No. P04-15500) supplemented with 5% FBS (Fetal Bovine Serum) (PAN-Biotech, Cat. No. P40-37500), 1% penicillin-streptomycin (Pen/Strep) (PAN-Biotech, Cat. No. P06-07100), 10 mmol HEPES buffer pH 7.4–7.6 (Roth, Cat. No. 6763.2), 5 μg/mL insulin (PAN-Biotech, Cat. No. P07-04300) and 1 μg/mL hydrocortisone (Sigma-Aldrich, Cat. No. H0888-1G). The MDA-MB-231 and MDA-MB-468 cells were cultured in DMEM medium (PAN-Biotech, Cat. No. P04-04510) supplemented with 10% FBS, 1% Pen/Strep and 20 mM HEPES. The primary cell culture UIVC-IDC-4 was isolated from patients from Mexico as previously described (Espinoza-Sánchez et al., 2017; Espinoza-Sánchez et al., 2018; Lango-Chavarría et al., 2017) and were cultivated in EpiCult-C Medium Kit (STEMCELL, Cat. No. 05630) supplemented with 10% FBS low toxin (PAN-Biotech, Cat. No. P30-3302), 1% Pen/Strep, 2 mM L-glutamine (PAN-Biotech, Cat. No. P04-80100) and 0.48 μg/mL hydrocortisone. Additionally, a non-malignant breast epithelial cell line, MCF10A and a non-cancerous keratinocyte cell line, HaCaT were used to test the toxicity of largazole on non-cancerous cells. MCF10A cells were cultured in DMEM/F12 (PAN-Biotech, Cat. No. P04–41150) supplemented with 5% horse serum (Invitrogen, Cat. No. 16050–122), 1% Pen/Strep, 20 ng/mL EGF (Preprotech, Cat. No. 315–09), 0.5 μg/mL hydrocortisone, 100 ng/mL Cholera Toxin (Sigma, Cat. No. C-8052), 10 μg/mL insulin, and 25 mM HEPES. HaCaT were cultured in DMEM containing 10% FBS, 1% Pen/Strep solution, and 25 mM HEPES buffer. All cells were cultured in a 90% humidified atmosphere with 5% CO_2_ at 37  C.

For further experiments, the cells were washed with phosphate-buffered saline (PBS, 137 mmol NaCl, 2.7 mM KCl, 10 mM Na2HPO4, 1.8 mM KH2PO4) and detached by adding Accutase (PAN-Biotech, Cat. No. P10-21100) for 5 to 10 min, depending on the cell line. The reaction was stopped by adding twice the volume of the standard culture medium to the cells, which were then collected in a 15 mL centrifuge tube. Cell number was determined by staining with 4′,6-diamidino-2-phenylindole DAPI (Sysmex Partec, Cat. No. 05-5001), and flow cytometric analysis (CyFlow Space, Partec, Münster, Germany) was performed. The data were visualized using FloMax software (Quantum Analysis, Münster, Germany).

If not otherwise specified, cells were treated with largazole, synthesized similarly as described (Ying et al., 2008; Chen et al., 2018) or DMSO as control for 24 h. Cells were then further analyzed.

### 2.2 MTT assay (3-[4,5-dimethylthiazol-2-yl]-2,5 diphenyl tetrazolium bromide assay)

The metabolically active cell population was determined using 3-(4,5-dimethyl-2-yl)-2,5-diphenyltetrazolium bromide (MTT, Sigma-Aldrich, Cat. No. M2128). 4,000 cells/well (MDA-MB-231, MDA-MB-468, SUM149PT and UIVC-IDC-4), 6,000 cells/well (HaCaT) or 10,000 cells/well (MCF10A) were seeded in a 96-well plate. Later, the cells were treated with different concentrations of largazole in a range of 0.1 to 5000 nM to determine the IC50 value. In chemoresistance assays, they were treated with largazole in combination with cisplatin or paclitaxel in the range of 1 nM–10 mM or 10 pM - 5 nM. After 96 h, MTT solution was added to the media according to the manufacturer’s protocol. After 24 h of incubation, the plates were analyzed using a plate reader (TriStar LB942, Berthold Technologies), measuring absorbance at 570 nm and 650 nm. Normalization was performed on the controls and data were plotted on a logarithmic scale for the individual dose points. For subsequent analysis, sublethal concentrations of largazole were used, where approximately 50%–70% of the untreated metabolically active cell population remained. Therefore, for further experiments, the concentration of largazole was 30 nM for the SUM149PT cells, 50 nM for MDA-MB-231, 100 nM MDA-MB-468, and 20 nM for the primary cells.

### 2.3 Cell cycle

The cells were seeded as described above. Cells were harvested and stained with DAPI after 24 h of largazole/DMSO treatment. Fluorescence intensity was analyzed using a flow cytometer (CyFlow Space, Partec) by measuring excitation via a UV diode laser at 365 nm and emission at 455 nm. The cell cycle profile was analyzed based on approximately 30,000 individual cells (FloMax, Quantum Analysis).

### 2.4 Trypan blue staining

Cells were treated with the largazole for 24 h. Afterwards, cells were harvested and stained with Trypan blue at a ratio of 1:2 for 3 min. The cell suspension was transferred onto a slide, and four images per sample were acquired using a microscope (ZEISS Primovert). For analysis, cells were counted using the Images J counting tool and classified as dead or viable based on the staining color.

### 2.5 BrdU assay

500,000 cells were treated with largazole or DMSO for 24 h. Afterwards, the supernatant was discarded and new media containing BrdU reagents 2′-Deoxycytidine hydrochloride (Merk, Cat. No. D8006) and 5-Bromo-2′deoxyuridine (Serva, Cat. No. 15240) were added to the cells for 30 min. The cells were harvested with Accutase, washed, and centrifuged. The supernatant was discarded, and the pellet was resuspended in ice cold ethanol (70%) and stored at −20 °C until further use. For the measurement, the cells were washed and resuspended in pepsin-HCl (Sigma Aldrich, Cat. No. P6887). Afterwards, the cells were washed, centrifuged, and resuspended in 100 µL 0.1% BSA/PBS containing anti BrdU-FITC antibody (BioLegend, Cat. No. 364104). Cells were washed and resuspended in propidium iodide solution for 20 min. Measurement was performed on a flow cytometer (CyFlow space, Partec) and the data was visualized using FloMax software (Quantum Analysis).

### 2.6 Digital holographic microscopy (DHM)

DHM was applied using a DHM microscope as previously described in detail by Eder et al. (2022). Cells were seeded in a 24-well plates (Ibidi, Cat. No. 82426) and treated with largazole. Series of DHM quantitative phase images were acquired every 15 min for 48 h for each observed well/field of view (FOV) from which subsequently the temporal dependency of the cellular dry mass in each FOV was calculated (Eder et al., 2022). In addition, the acquired time-lapse series of DHM quantitative phase images were evaluated for cell motility utilizing custom built software for retrieval of the individual single cell migration trajectories (Kemper et al., 2010), from which subsequently the parameters Euclidian distance, total distance and mean velocity were determined (for details see (Wischmann et al., 2023)).

### 2.7 RealTimeGlo–tracking the metabolically active cell population over Time

The cells were harvested and 4,000 cells/well were seeded in a 96-well plate. The reagents were then brought to 37 °C and diluted into the media with defined amounts of MT Cell Viability Substrate and NanoLuc Enzyme (Promega - RealTime-Glo™ MT Cell Viability Assay). The mixture with the reagents and different concentrations of largazole was added to the cells. The metabolically active cell population was measured for up to 72 h using a plate reader (TriStar LB942, Berthold Technologies) via luminescence.

### 2.8 HDAC-Glo

The cells were harvested, and 4,000 cells/well were seeded into a 96-well plate. Cells were treated for 3 h with either varying concentrations or a specific concentration of largazole. After 3 h, the HDAC-Glo™ I/II reagent (HDAC-Glo™ I/II Assays and Screening System, Promega, Cat. No. G6420) was prepared and added to the cells. The plate was incubated for 15–45 min. Afterwards, the luminescence was measured in different biological replicates using a plate reader (TriStar LB942, Berthold Technologies).

### 2.9 CD24/CD44 assay

Cells were treated with largazole, harvested using Accutase, and 500,000 cells were used for antibody staining of CD24 PE (BD Pharmingen, Cat. No. 555428), CD44 APC (BD Pharmingen, Cat. No. 559942) and corresponding isotype controls (all from BD Pharmingen, Franklin Lakes, NJ, USA, Cat. No. 555576 and 345816). All experiments were performed according to the manufacturer’s protocol. Measurement was performed on a flow cytometer (CyFlow space, Partec) and the data was visualized using FloMax software (Quantum Analysis).

### 2.10 Annexin V assay–apoptosis

The cells were seeded in six-well plates and incubated overnight. Cells were then treated with largazole or DMSO for 24 h. Cells were harvested, counted and stained with the BD Pharmingen™ FITC Annexin V Apoptosis Detection Kit I (BD Biosciences, Cat. No. 556547) as described in the manufacturer’s protocol. Measurement was performed on a flow cytometer (CyFlow space, Partec) and the data was visualized using FloMax software (Quantum Analysis).

### 2.11 Mammosphere formation

SUM149PT cells have been shown to form mammospheres (Wolf et al., 2013), whereas MDA-MB-231 and MDA-MB-468 cells lack this capability (Manuel Iglesias et al., 2013). To evaluate the self-renewal and regenerative capacity of the cells, we conducted mammosphere formation assays in both primary and secondary generations using SUM149PT cells and primary breast cancer cultures. Equal numbers of control and largazole-treated cells were seeded (10,000 cells/mL) into six-well plates (Greiner Bio-One, Cat. No. 657 185) in 3D Tumorsphere Medium XF (PromoCell, Cat. No. C-28070). After 7–10 days, the primary spheres were imaged, and their number and maximum diameter were quantified using ImageJ software. Subsequently, cells were enzymatically dissociated, re-seeded as single cells into 96-well plates under identical conditions, and cultured for an additional 14 days. Secondary spheres were then imaged and analyzed. In both generations, structures exceeding 150 μm in diameter were classified as mammospheres.

### 2.12 miRNA isolation and transfection

miRNA was isolated with the mirVana™ Kit from Ambion™ (ThermoFisher Scientific, Cat. No. AM1561) according to the manufacturer’s instruction. Briefly, the cells were harvested and washed. The pellet was resuspended in 500 µL Lysine/Binding-Buffer and 50 µL miRNA Homogenate Additive was added to the mixture, vortexed and incubated on ice. Afterwards, 500 µL phenol/chloroform-solution (Invitrogen, Cat. No. AM9720) was added and vortexed for 30–60 s. The samples were then centrifuged, and the aqueous phase was carefully transferred into a new tube. Now, the regular isolation of miRNA started. Therefore, 100% EtOH was added with a final volume of 1/3. The solution was then transferred onto a filter and centrifuged. The flowthrough was collected and 2/3 of the volume was mixed with 100% EtOH. The mix was then placed on a new filter and centrifuged. The supernatant was discarded, as the miRNA was attached to the filter. The filter was washed with 700 µL miRNA wash solution #1 and centrifuged. The flowthrough was discarded, and the filter was washed twice with 500 µL wash solution #2/3. Finally, the miRNA was eluted with 100 µL Elution solution (preheated to 95 °C) and centrifuged. Now the concentration was determined via absorption. The TaqMan® MicroRNA Reverse Transcription Kit (ThermoFisher Scientific, Cat. No. 4366596) was used for reverse transcription of 100 ng microRNA with miR-specific primer systems according to the kit’s guidelines. For further analysis of the miRNA, a qPCR was performed by using TaqMan® assays. U6 was used as a reference. Analyses were run on a Rotor-Gene Q machine (Qiagen). Data were expressed as fold change using the 2^–ΔΔCT^ method.

The miRNA transfection was performed similar to Wischmann et al. (2023). Briefly, cells were seeded in a 6-well plate (Greiner, Solingen, Germany) 24 h prior to treatment. Subsequently, transfection was performed with 2 µL Lipofectamine RNAiMAX (Invitrogen, ThermoFisher Scientific, Cat. No. 13778075) mixed with OptiMEM media (Gibco, Cat. No. 31985062) with the addition of 2 µL hsa-miR-125b-5p (ThermoFisher Scientific, Assay ID PM10148), anti-hsa-miR-125b-5p (ThermoFisher Scientific, Assay ID AM10148), or negative control (ThermoFisher Scientific, Assay ID AM17111) (10 pM) per well. 24 h later, transfection reagents were replaced by regular medium.

### 2.13 Cell lysis and immunoblot analysis

Procedures were performed as previously described (Haiduk et al., 2023). Cells were washed, harvested, and the pellet was stored at −20 °C. For the lysis the pellet was resuspended in RIPA-Buffer (20 mM Tris HCl, 137 mM NaCl, 1% Triton X-100, 2 mM EDTA) supplemented with 10 mM NaF, 10 mM β-glycerolphosphate, 1 mM Na_3_VO_4_ and 10% inhibitor cocktail (SigmaFast TM, Sigma-Aldrich, Cat. No. B5655). The protein concentration was determined photometrically (Biophotometer, Eppendorf) using the Bradford assay (Sigma-Aldrich, Cat. No. B6916). Protein separation was performed by SDS-PAGE (GE Healthcare, Cat. No. 10600001), followed by the semi-dry blotting technique (Trans-Blot Turbo Transfer System, Bio-Rad, 25 V, 2.5 A for 10 min) and blocking of non-specific binding sites (5% BSA solution in TBS). The primary antibody was applied overnight at 4 °C against MSI1 (Santa Cruz, Cat. No. sc-135721), MSI2 (Abcam, Cat. No. ab76148), Notch 3 (Cell Signaling Cat. No. #5276S), NUMB (Santa Cruz, Cat. No. sc-136554), p21 (Cell Signaling, Cat. No. 2947S), and TERT (Invitrogen, Cat. No. PA511446). ß-Actin (Sigma Aldrich, Cat. No. A5441) was used as a loading control. Specific secondary antibodies (R&D Systems) were diluted in 5% BSA in TBS-T (10 mM Tris HCl, 150 mM NaCl, 0.2% Tween®20) and incubated for 1 h at room temperature. Chemiluminescence was induced by the application of Super Signal^TM^ West Pico PLUS chemiluminescent substrate (Thermo Fisher Scientific, Cat. No. 34580), which was then visualized using FUSION SL (Vilber Lourmat, Marne-la-Vallée, France). Data were analyzed using ImageJ software (National Institute of Health, Bethesda, USA)

### 2.14 RNA sequencing

For RNA sequencing, the primary cells were treated with largazole or DMSO for 24 h. The cells were then harvested and processed for RNA isolation. Notably, an RNase inhibitor was added to the protocol. The pure RNA was then analyzed by the Core Facility Genomics of the Medical Faculty of the University of Münster using the Illumina NextSeq 2000 system. The bioinformatics analyses were performed similar to previous study (Brücksken et al., 2025; Zhao and Korsching, 2025). DESeq2 data were imported and processed using RStudio software (v2024.09.0+374). A cancer hallmarks analysis was conducted as a gene ontology (GO) enrichment analysis using significantly dysregulated transcripts (p < 0.05) in the CancerHallmarks tool (Menyhart et al., 2025). Additionally, a gene set enrichment analysis (GSEA) was performed using the Broad Institute’s GSEA tool (Mootha et al., 2003; Subramanian et al., 2005), with data processed according to the recommended guidelines. Normalized counts from the DESeq2 analysis, adjusted to the required format, were used as input for GSEA.

### 2.15 Colony formation with irradiation

The cells were treated with a defined concentration of largazole or DMSO as control for 24 h. The cells were then irradiated with a TrueBeam linear accelerator (Varian, Palo Alto, USA) with 2, 4 or 6 Gy at a dose rate of 4.8 Gy per minute. Afterwards, the cells were harvested, counted and seeded in predefined numbers in a 6-well plate. The cells were further treated with largazole and 5%–10% more FBS (depending on the cell line) was added to the media. The medium was changed every three to 4 days. After 7–14 days the cells were washed and fixed with Loeffler methylene blue staining solution (0.6% Methylene blue, 21% ethanol, 0.01 NaOH) for 90 min. Groups of more than 50 cells were defined as colonies and counted microscopically. The plating efficiency (PE) was calculated as

$$PE=\frac{Number\;of\;colonies}{Number\;of\;seeded\;cells\;}.$$

Subsequently the PE of irradiated cells was normalized to the PE of non-irradiated cells to calculate survival fractions (SF). SFs were calculated separately for largazole-treated cells and controls:

$$SF=\frac{PE\;\left(irradiated\;cells\right)}{PE\;\left(unirradiated\;cells\right)}.$$

SFs of largazole-treated cells and controls were then compared. This standard radiation biology analysis ensures that only a synergistic effect (beyond additive, separate effects of largazole treatment and irradiation) will show a statistically significant difference.

### 2.16 Gamma-H2AX DNA double strand breaks

The cells were treated for 24 h and irradiated with 4 Gy. Afterward the cells were harvested and fixed at defined time points (30, 60, 120, 240, 360, and 1,440 min) after irradiation with 70% iced cold ethanol and stored at −20 °C. For the staining, the cells were centrifuged, permeabilized with 0.25% Triton X-100 and blocked with 5% FBS. Next the cells were stained with the primary antibody against mouse Anti-phospho-histone H2AX (Ser139) (Millipore/Merck, Cat. No. 05-636), followed by the secondary antibody Alexa Fluor^TM^ 488 goat anti-mouse (Invitrogen, Cat. No. A11001). Finally, the cells were stained with propidium iodide. Flow cytometric analysis was performed with a red 635 nm and a blue 488 nm laser and FloMax software (Quantum Analysis) for data acquisition and evaluation.

### 2.17 Immunocytochemistry – 53BP1 staining

A 24-well plate was prepared with 13-mm glass coverslips and 30,000 cells were seeded into each well. Subsequently, the cells were treated with largazole for 24 h at 37 °C and 5% CO2 and irradiated with 4 Gy. After 30 min, the cells were washed three times with PBS and fixed with 4% Paraformaldehyde (PFA) in PBS for 20 min at room temperature. The PFA was discarded and the coverslips were washed three times with PBS. Next, the blocking and permeabilization buffer was applied for 1 h under gentle shaking, followed by the incubation with the primary antibody (53BP1, Novus, Cat No. NB100-305) overnight at 4 °C under shaking conditions. The primary antibody was removed, and the cells were washed three times before incubation with the secondary antibody (AlexaFluor488 donkey anti-rabbit IgG (H+L), ThermoFisher, Cat. No A32790) for 1 h at room temperature. The coverslips were washed again, and the nuclei were stained with 50 μg/mL Hoechst (Hoechst 33342, ThermoFisher, Cat No. H21492) for 10 min. Afterwards, the coverslips were washed and mounted onto slides using Fluoromount Aqueous mounting medium (Siegma-Aldrich, Cat No. F4680). The samples were analyzed by fluorescence microscopy (Axiovert 5, Zeiss). Four to five randomly selected images were acquired using 20x objective. The images were analyzed by using ImageJ and Foci Analyzer.

### 2.18 Statistics

All *in vitro* experiments were repeated at least three times in independent technical and experimental test series. Statistical analysis was performed with Graph Pad Prism 8 (GraphPad software, La Jolla, CA, USA) using Student's t-test to demonstrate the significance of differences between treatments. The individual p-values are indicated above the compared data. Multiple test correction was performed according to Benjamin-Hochberg/FDR. The significance level was defined as p < 0.05 (*: p < 0.05; **: p < 0.01; ***: p < 0.001; ****: p < 0.0001).

## 3 Results

### 3.1 Largazole reduces TNBC cell growth and the metabolically active cell population in a dose-dependent manner

**FIGURE 1:**
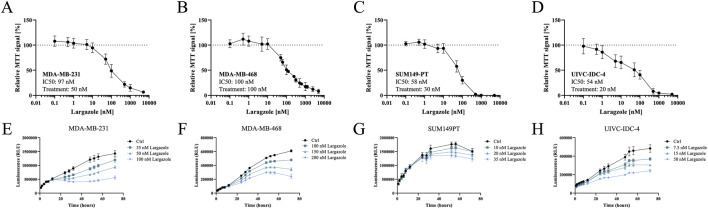
Effects of largazole on TNBC cell metabolism. Largazole-associated effects on the metabolically active cell population in TNBC cell lines MDA-MB-231, MDA-MB-468, SUM149PT and primary cell culture UIVC-IDC-4. The cells were treated for 96 h using different concentrations of largazole. The largazole tolerance differed, representing changes in the remaining metabolically active cell population, as measured via MTT Assays and calculated as IC50 values: in **(A)** MDA-MB-231, **(B)** MDA-MB-468, **(C)** SUM149PT, and **(D)** UIVC-IDC-4. In luminescence-based cell viability assays, largazole showed a concentration-dependent reduction in the metabolically active cell population over time in all tested cell lines and cultures **(E)** MDA-MB-231, **(F)** MDA-MB-468, **(G)** SUM149PT and **(H)** in cell culture UIVC-IDC-4. All experiments were conducted in at least three independent replicates. Data are presented as mean ± standard deviation, as indicated by the error bars.

To determine optimal treatment concentrations, we performed an MTT assay using different concentrations of largazole for 96 h. The results showed a dose-dependent reduction in the metabolically active cell population across all tested cell lines (Figures 1A–C) and primary triple-negative breast cancer (TNBC) cell culture (Figure 1D). The primary cell culture UIVC-IDC-4 exhibited the lowest IC50 value of 54 nM followed by SUM149-PT with 58 nM, MDA-MB-231 with 97 nM and MDA-MB-468 with 100 nM. For comparison, we also treated non-cancerous skin keratinocytes (HaCaT) and benign breast epithelial cells (MCF10A) with largazole. In HaCaT cells, the calculated IC50 value was 3 μM, indicating a much lower sensitivity to the inhibitor compared to TNBC cells. For MCF10A cells, an IC50 value could not be determined as the metabolically active cell population remained at 65% of untreated levels at 5 µM of largazole, the highest tested concentration, and 50-fold the IC50 concentration of the least-sensitive breast cancer culture (Supplementary Material S1, Supplementary Figure S1).

To assess the effect of largazole on the metabolically active cell population over time, we conducted a longitudinal luminescence-based viability assay on TNBC cells over a period of at least 72 h. Cells were treated with different concentration of the inhibitor, and results showed a sustained, concentration-dependent reduction of the metabolically active cell population throughout the observation period (Figures 1E–H).

For subsequent analyses, concentrations of largazole at which approximately 50%–70% of metabolically active cell population was retained were chosen, based on MTT assay results.

### 3.2 Largazole impairs clonogenicity, inhibits DNA-synthesis and alters the cell cycle in TNBC

**FIGURE 2:**
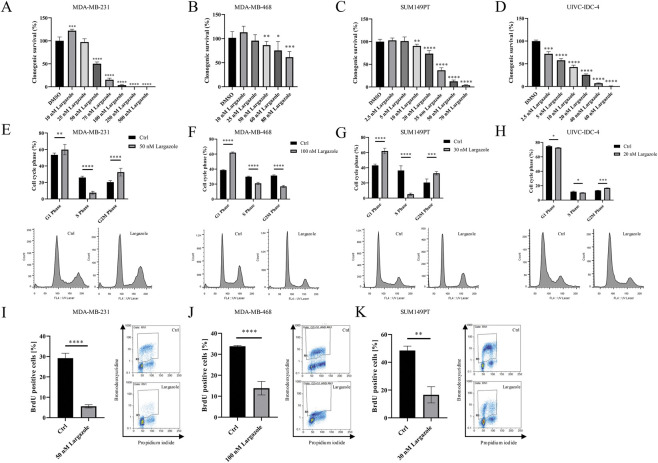
Largazole induced changes in colony formation, cell cycle and DNA synthesis. Changes in colony formation, cell cycle and DNA synthesis were induced by largazole. Largazole treatment reduced colony forming ability at increasing concentrations, representing in **(A)** MDA-MB-231, **(B)** MDA-MB-468, **(C)** SUM149PT, and **(D)** UIVC-IDC-4. Clonogenic survival in % relative to the DMSO control is shown. The effects of largazole after 24 h leads to cell cycle alterations in **(E)** MDA-MB-231, **(G)** SUM149PT, **(F)** MDA-MB-468, and **(H)** the primary cell culture UIVC-IDC-4. Representative measurements are also shown. DNA synthesis visualized in BrdU assays was reduced in **(I)** MDA-MB-231 cells, **(J)** MDA-MB-468 cells, and **(K)** SUM149-PT cells after largazole treatment. All experiments were conducted in at least three independent replicates. Data are presented as mean ± standard deviation, as indicated by the error bars.

Colony formation assays were performed to investigate the effects of largazole on clonogenicity of breast cancer cells. Singularized cells were seeded and treated with different concentrations of largazole. The clonogenic ability of all tested cell lines and primary cultures was significantly reduced in a concentration-dependent manner (Figures 2A–D).

In cell cycle studies, we observed an induction of cell cycle arrest, although the specific alterations varied across cell lines and primary cultures after 24 h of largazole treatment. In the MDA-MB-231 and SUM149-PT cell lines, there was a significant reduction in the S phase (p < 0.0001, and p < 0.0001, respectively), accompanied by an increase in both the G1 phase (p = 0.0003, p < 0.0001) and G2/M phases (p < 0.0001, p = 0.0002) (Figures 2E,G). In MDA-MB-468 cells, the fractions of cells in the S phase (p < 0.0001) and G2/M phase (p < 0.0001) were decreased, while the G1 phase (p < 0.0001) was increased (Figure 2F). In the primary cell line UIVC-IDC-4, fewer cells entered the S phase (p = 0.0474) and G1 phase (p = 0.0253) when treated with largazole, whereas the proportion of cells in the G2/M phase increased significantly (p = 0.0001) compared to the control (Figure 2H). Hence, all tested breast cancer cell lines and primary culture showed a significant, treatment-induced reduction in the proportion of S phase cells.

Consistent with the observed reduction in S phase, all examined cell lines showed a significant decrease in the DNA synthesis, as measured by bromodeoxyuridine (BrdU) assay (Figures 2I–K).

### 3.3 Largazole reduces proliferation and cell motility

**FIGURE 3:**
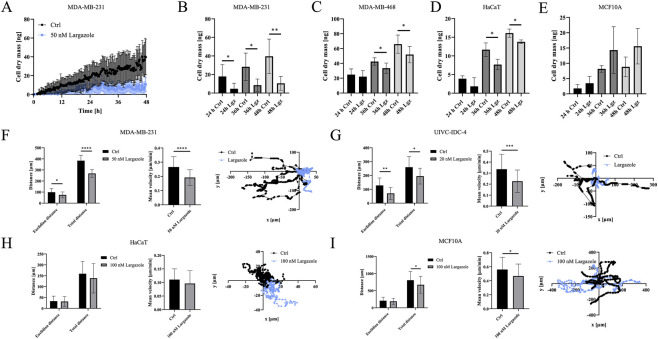
Largazole reduces cell proliferation and motility. Development of cell dry mass and motility were monitored via digital holographic microscopy (DHM). Largazole treatment led to a decreased cell dry mass increment over time in MDA-MB-231 (representative graph in **(A)**, statistical assessment for 24, 36, and 48 h timepoints in **(B)**), MDA-MB-468 **(C)**, HaCaT **(D)**, and MCF10A **(E)** cells. Largazole-treated cells of **(F)** MDA-MB-231 and **(G)** primary cell culture UIVC-IDC-4 showed significantly reduced Euclidian distance, total distance and mean velocity. Effects were less pronounced in non-malignant **(H)** HaCaT and **(I)** MCF10A cells. All experiments were conducted in at least three independent replicates. Data are presented as mean ± standard deviation, as indicated by the error bars.

Digital holographic microscopy was employed to assess the impact of largazole on cell proliferation and motility over a 48 h period. Largazole treatment led to a time-dependent decrease in dry mass accumulation in both MDA-MB-231 (48 h p = 0.005) and MDA-MB-468 (48 h p = 0.045) cell lines, indicating reduced proliferation (Figures 3A–C), though effects were moderate in MDA-MB-468. Due to the very thin cell thickness, accompanied with a low signal to noise ratio in DHM quantitative phase images, the cellular dry mass could not be reliably quantified in the primary UIVC-IDC-4 cell culture, though there was a visually confirmed decrease in proliferation after largazole application in time-lapse videos (Supplementary Material S1; Supplementary Figure S2; Supplementary Material S2). Time-lapse videos of all tested cultures, UIVC-IDC-4, MDA-MB-231 and MDA-MB-468 pointed towards a reduced frequency of cell division (Supplementary Material S2). Conversely, MCF10A cells showed no effects on cell dry mass accumulation while HaCaT cells only showed a slight decrease (Figures 3D,E).

In dedicated flow cytometric apoptosis measurements we found that the observed reduction of dry mass increment in digital holographic microscopy is not caused by cell death, as only MDA-MB-231 cells showed an increase in the late apoptotic/necrotic cell proportion (p = 0.001). Conversely, most other cell lines showed a slight decrease in apoptosis after largazole application (Supplementary Material S1; Supplementary Figure S3). Trypan blue measurements performed 24 h after largazole treatment supported these findings with no changes or a very modest increase in living cell proportions after largazole treatment (Supplementary Material S1, Supplementary Figure S4).

To evaluate changes in cell motility, single cell tracking analysis was performed. Both the MDA-MB-231 and the primary culture UIVC-IDC-4 showed significant reduction in Euclidean distance (p = 0.0289 and p = 0.0082, respectively), total distance (p < 0.0001, p = 0.0285), and mean velocity (p < 0.0001, p = 0.0004) by largazole treatment (Figures 3D,E). Notably, MDA-MB-468 cells showed no meaningful baseline motility (data not shown). Consequently, no differences in cell motility were observed.

### 3.4 Largazole reduces stem cell characteristics in TNBC

**FIGURE 4:**
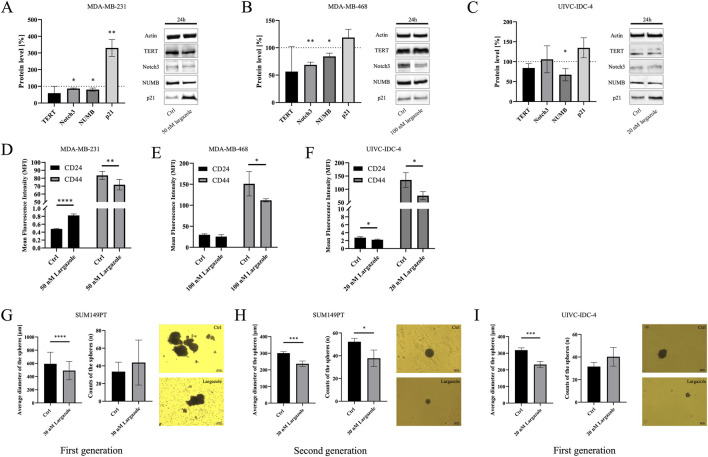
Largazole-induced changes in stem cell characteristics. Protein level changes 24 h after largazole treatment differed among the different cell types. In MDA-MB-231 **(A)** and MDA-MB-468 **(B)** protein levels of Notch3 and NUMB were reduced while p21 levels were increased. In UIVC-IDC-4 cells **(C)** protein levels of NUMB were decreased. To test largazole effects on stem cell characteristics, changes in surface markers CD24 and CD44 were analyzed via flow cytometry 24 h after treatment. In MDA-MB-231 the expression of stemness-associated surface marker CD24 increased, while CD44 decreased significantly **(D)**. MDA-MB-468 cells presented a significant lower expression of CD44 after largazole treatment **(E)** and the primary cell culture UIVC-IDC-4 showed a significant reduced expression of CD44 as well as CD24 **(F)**. Additionally, in SUM149-PT and UIVC-IDC-4 cells largazole led to a significant reduction of the average diameter of the spheres in first generation **(G,I)** and reduced diameter and count of the spheres in the second generation of SUM149-PT **(H)** in mammosphere formation assays. Next to the diagrams sample images are presented (300 µm scale in the lower right corner). All experiments were conducted in at least three independent replicates. Data are presented as mean ± standard deviation, as indicated by the error bars.

Western blot analysis of stemness-associated proteins indicated that largazole treatment reduced stem cell-like properties in TNBC cells. In both MDA-MB-231 and MDA-MB-468 cells, protein levels of Notch3 (p = 0.0215) and Numb (p = 0.0289) were significantly reduced while MDA-MB-231 cells also show a significant increase in the cyclin dependent-kinase inhibitor p21 (p = 0.0029). In the UIVC-IDC-4, the protein levels of Numb were decreased (p = 0.0235) (Figures 4A–C).

Surface marker analysis by flow cytometry revealed further stemness-related changes. CD44^+^/CD24^-/low^ expression is commonly associated with breast cancer stem cells (Greve et al., 2012). In MDA-MB-231 cells, CD24 expression increased significantly (p < 0.0001) while CD44 was reduced (p = 0.0062) after largazole treatment (Figure 4D). CD44 levels were also reduced in MDA-MB-468 (p = 0.0165) (Figure 4E). In UIVC-IDC-4, both CD24 (p = 0.0376) and CD44 (p = 0.0316) expression levels were significantly decreased (Figure 4F).

To further assess cancer stemness, we evaluated the effects of largazole on sphere formation in SUM149-PT and UIVC-IDC-4 cells. In both cell types, the average diameter though not the number of the spheres in the first generation was significantly reduced (SUM149-PT p < 0.0001, UIVC-IDC-4 p = 0.0003). In the second generation, SUM149-PT cells exhibited a significant reduction in both average diameter (p = 0.0005) and total sphere counts (p = 0.0102) (Figures 4G–I). UIVC-IDC-4 cells did not show a meaningful sphere growth in the second generation.

### 3.5 Largazole inhibits HDAC activity and attenuates MSI expression through increased expression of miRNA-125b-5p

**FIGURE 5:**
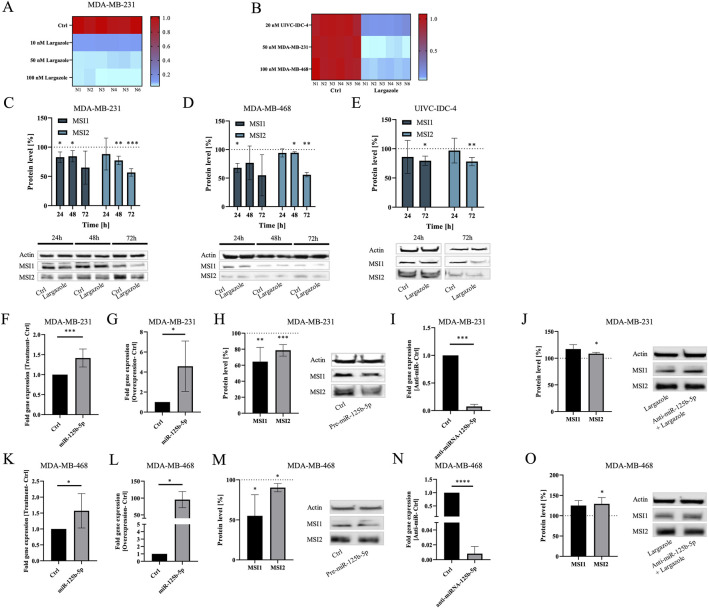
Effects of largazole on HDAC activity and miRNA-125b-5p/Musashi axis. A heatmap based on data from luminescence assays of HDAC activity demonstrates that in MDA-MB-231 largazole inhibited HDAC in a dose-dependent manner, consistent across biological replicates (columns) **(A)**. The test is sensitive for HDAC classes I and II. At previously defined treatment concentrations, largazole lowered the activity of HDACs in all tested cell cultures, again consistent across biological replicates (columns) **(B)**. Western blot analysis demonstrated that largazole treatment resulted in a time-dependent reduction of MSI1 and MSI2 protein levels in all tested cell lines and the primary cell culture with 100% representing control expression levels in the graphs **(C–E)**. qRT-PCR analysis of miRNA-125b-5p in largazole-treated MDA-MB-231 and MDA-MB-468 cells showed a significant increase in expression compared to the controls **(F,K)**. We subsequently overexpressed miR-125b-5p in MDA-MB-231 and MDA-MB-468 wildtype cells and confirmed overexpression via qRT-PCR **(G,L)**. miRNA-125b-5p-overexpressing MDA-MB-231 and MDA-MB-468 cells showed a relevant reduction of MSI1 and MSI2 protein expression with 100% representing control expression levels in the graphs **(H,M)**. We also downregulated miR-125b-5p using a specific anti-miR in MDA-MB-231 and MDA-MB-468 cells and validated the downregulation via qRT-PCR **(I,N)**. When comparing Musashi protein expression between largazole-treated cells and cells treated with both largazole and anti-miR-125b-5p, we found that Musashi protein levels were increased after combined treatment (100% represents expression after single treatment in the graphs) **(J,O)**.

HDAC activity was assessed in different TNBC cell lines and primary cell culture using a luminescence assay, comparing largazole-treated cells to DMSO-treated controls. We first confirmed a dose-dependent association between HDAC inhibition and largazole treatment in MDA-MB-231 cells (Figure 5A). We then determined that largazole consistently strongly reduced HDAC activity in all tested cell types at the previously defined treatment concentrations (Figure 5B). The assay measured HDAC class I and II activity, while the class I selectivity of largazole is well established (Hong and Luesch, 2012).

Western blot analysis of MSI1 and MSI2 protein levels at different times of largazole treatment revealed a time-dependent reduction in both proteins across all tested cell lines and primary culture (Figures 5C–E).

Literature suggested that HDAC inhibition might upregulate miR-125b-5p levels (Hsieh et al., 2015), a known suppressor of the Musashi proteins (Forouzanfar et al., 2021). We assessed this possible interaction in MDA-MB-231 and MDA-MB-468 cells. In largazole-treated MDA-MB-231 and MDA-MB-468 cells, miRNA-125b-5p expression was significantly increased compared to controls (MDA-MB-231: p = 0.0009, MDA-MB-468 p = 0.05) at effective HDAC-inhibitory concentrations of largazole (Figures 5F,K), further suggesting a downstream functional link. To additionally test this hypothesis, we overexpressed miR-125b-5p in MDA-MB-231 and MDA-MB-468 cells (MDA-MB-231: 4-fold, p = 0.0128, Figure 5G; MDA-MB-468: 100-fold, p = 0.02, Figure 5L) and observed a significant decrease in both MSI1 (MDA-MB-231: p = 0.0044; MDA-MB-468: p = 0.04) and MSI2 (MDA-MB-231: p = 0.0007; MDA-MB-468: p = 0.03) protein levels by Western blot analysis (Figures 5H,M).

To evaluate whether largazole regulated Musashi protein levels in a miR-125b-5p-dependent manner, we next assessed whether the absence of the miR-125b-5p interfered with the largazole-induced downregulation of the Musashi proteins. For this, we used a specific anti-miR-125b-5p that downregulated miR-125b-5p levels to < 10% residual expression in TNBC cells (MDA-MB-231: p = 0.0006; MDA-MB-468: p < 0.0001; Figures 5I,N). When comparing largazole treatment with largazole + anti-miR-125b-5p treatment, we found increased Musashi protein expression after combination treatment in both cell lines, suggesting that anti-miR-125b-5p abrogated largazole-induced downregulation of Musashi expression (Figures 5J,O).

### 3.6 Largazole causes a decrease in the expression of genes essential for cell cycle progression, DNA synthesis and double strand break repair

**FIGURE 6:**
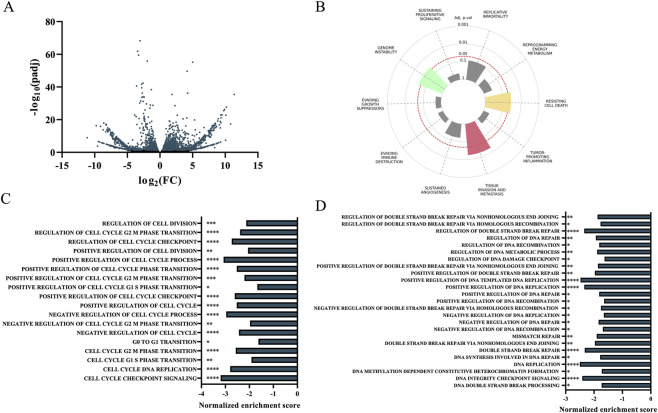
RNA sequencing results of the largazole-treated primary TNBC cell culture UIVC-IDC-4 compared to DMSO-treated controls. In a volcano plot, effects on gene expression are displayed for UIVC-IDC4 **(A)**. The observed clustering of targets is caused by a low baseline gene expression. Changes in signaling after largazole treatment was assessed for cancer-defining features using the Cancer Hallmarks Tool by inputting significantly dysregulated genes between groups (https://cancerhallmarks.com/) **(B)**. Changes in expression of particular pathways were shown for cell cycle progression **(C)** and DNA repair **(D)** in UIVC-IDC4 cells using GSEA.

After confirming the largazole/miR-125b-5p/MSI pathway in MDA-MB-231 and MDA-MB-468 cells, we were interested in assessing broader largazole-associated gene expression changes. We subsequently performed RNA sequencing in the primary cell culture UIVC-IDC-4 24 h after largazole treatment at 20 nM (Figure 6A). Significantly dysregulated genes found were analyzed using the Cancer Hallmarks tool to identify overrepresented cancer hallmarks. Largazole caused a significant dysregulation of genes relevant for “genome instability” (OR = 1.495, p = 0.036), “tissue invasion and metastasis” (OR = 1.318, p = 0.0176), and “resisting cell death” (OR = 1.309, p = 0.0361, Figure 6B). Gene set enrichment analysis (GSEA) across all quantified genes underscored this result, demonstrating a strong and statistically significant decrease in cell cycle progression-associated signaling (Figure 6C), including a loss in “cell cycle checkpoint signaling”, “regulation of cell cycle G2 M phase transition”, and “regulation of cell division”. Analyses also revealed a strong and statistically significant attenuation of DNA repair-related pathways, including “double strand break repair”, “DNA replication”, and “positive regulation of DNA repair” (Figure 6D).

### 3.7 Largazole is a radiosensitizer in TNBC

**FIGURE 7:**
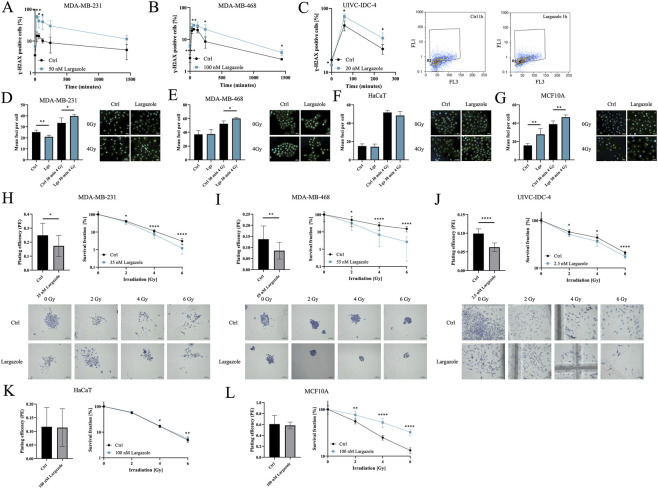
Largazole functions as a radiosensitizing agent in TNBC. Largazole treatment led to an increased radiosensitivity in triple negative breast cancer cells. In the tested cell lines **(A)** MDA-MB-231 and **(B)** MDA-MB-468 as well as **(C)** in the primary cell culture UIVC-IDC-4, largazole treatment led to a significant increase in DNA double strand breaks after irradiation compared to the controls tested via γ-H2AX measurements. Representative flow cytometric measurements for 1 h post-irradiation in UIVC-ICD-4 cultures are also shown; gating was consistent for all cell cultures and all timepoints. For additional validation, 53BP1 foci analyses were performed, both in unirradiated control- and largazole-treated cells as well as 30 min after 4 Gy irradiation in MDA-MB-231 **(D)**, MDA-MB-468 **(E)**, HaCaT **(F)**, and MCF10A **(G)** cells. After irradiation with 2, 4 and 6 Gy **(H)** MDA-MB-231 cells as well as **(I)** MDA-MB-468 cells and **(J)** primary cell culture UIVC-IDC-4 showed substantially reduced survival fractions (line graphs) in addition to a reduced plating efficacy in unirradiated cells (bar graphs). Non-malignant **(K)** HaCaT and **(L)** MCF10A showed increased survival fractions and no significant changes in plating efficiency. All experiments were conducted in at least three independent replicates. Data are presented as mean ± standard deviation, as indicated by the error bars.

To investigate the impact of largazole on the DNA damage response, TNBC cells were subjected to ionizing radiation and subsequently stained for γ-H2AX, a marker of DNA double-strand breaks. Cells treated with both largazole and radiation exhibited a higher number of γ-H2AX positive cells compared to controls immediately and multiple hours after irradiation (Figures 7A–C). For additional validation, a 53BP1 foci staining was performed 30 min after irradiation and the number of foci per cell was quantified. MDA-MB-231, MDA-MB-468, and MCF10A cells exhibited a higher number of foci per cell after combined largazole and irradiation treatment. Conversely, HaCaT cells showed no changes (Figure 7).

Finally, we performed clonogenic assays on irradiated cells to assess whether largazole sensitized cells to irradiation and attenuated cell survival. Largazole treatment resulted in a loss of plating efficacy in unirradiated TNBC cells (bar graphs, Figures 7H–J). In addition, largazole–at concentrations where class I HDAC activity is compromised–exhibited a consistent and meaningful radiosensitizing effect in both MDA-MB-231 and MDA-MB-468 cell lines, as well as in the primary cell culture, as evidenced by significantly reduced survival fractions after irradiation doses of 2, 4, and 6 Gy (line graphs, Figures 7H–J). Conversely, colony formation assays with the keratinocyte cell line HaCat and the healthy breast tissue cell line MCF10A indicated slightly (HaCaT) or strongly increased (MCF10A) cell survival after combined largazole and irradiation treatment compared to irradiation alone (Figures 7K,L).

## 4 Discussion

In this study, we found that largazole attenuates proliferation, migration and stemness in breast cancer cells. We confirmed that largazole downregulated the Musashi proteins, identifying a novel largazole/miR-125b-5p/Musashi axis at HDAC-inhibitory concentrations. Largazole treatment finally compromised DNA repair, increased irradiation-induced DNA double strand breaks and sensitized cells to irradiation.

### 4.1 Largazole attenuates triple-negative breast cancer cell clonogenicity and migration

Largazole is a potent class I HDAC inhibitor, discovered from marine cyanobacteria (Hong and Luesch, 2012). As is typical for HDAC inhibitors, it has been shown to meaningfully abrogate cancer cell viability in glioblastoma (Al-Awadhi et al., 2020), melanoma (Bowers et al., 2008), and colon cancer (Liu et al., 2010). In breast cancer, largazole cooperates with dexamethasone to reduce invasiveness through complementary mechanisms to induce and properly locate E-cadherin at the cell surface (Law et al., 2013). Our data corroborate antiproliferative effects against a panel of triple-negative breast cancer cell lines as well as a primary, patient-derived cell culture. Importantly, the reduction in the metabolically active cell population seen in MTT assays is likely primarily related to reduced proliferation as only small and anti-apoptotic effects were observed after largazole application in dedicated flow cytometric apoptosis measurements. The time-lapse digital holographic microscopy files support this hypothesis as cell division was visibly reduced. Finally, trypan blue measurements also indicate that cell death was not meaningfully changed, again with a very slight decrease in largazole-treated samples in some cultures.

Intriguingly, HaCaT and MCF10A cells exhibited more than 20-fold higher IC50 values compared to the TNBC cell cultures and showed very limited–if any–reduction in cell viability in longitudinal digital holographic microscopy measurements, suggesting that non-malignant keratinocytes and non-cancerous breast epithelial cells are significantly more resistant to the cytotoxic properties of largazole. This strong difference suggests that largazole may disproportionately affect cancer cells, an important prerequisite for therapeutic application. Accordingly, one largazole-inspired drug candidate, bocodepsin (OKI-179), reached Phase 1/2 clinical trials for solid tumors (Schreiber et al., 2023).

Moreover, the dose-dependent largazole-mediated effects appear to be long-lasting, as the attenuation in the metabolically active cell population persisted for at least 72 h after application. This indicates that while a previous colon cancer *in vivo* models utilized daily application of largazole (Liu et al., 2010) less stringent regimens may also be feasible.

Besides the reduced proliferation, we also observed a consistent cell cycle dysregulation with a reduction in S phase cells after largazole treatment. This suggests a reduced DNA synthesis, as also corroborated by the reduction in BrdU measurements. Notably, some cells remained in the BrdU-negative S phase in the largazole samples. However, they were substantially reduced in number when compared to the BrdU-negative S phase cells in the control samples. There were slight differences between cultures with regards to the other cell cycle phases: While the cell line data found an accumulation of cells in the G1 phase, a finding also previously suggested for colon cancer (Liu et al., 2010), primary UIVC-IDC-4 cells showed an increase in the G2/M phase instead, which was also found in urothelial cancer by Hoffmann et al. (2021).

Mechanistically, we observed an overexpression of p21 which was also found in colon cancer after largazole application (Hong and Luesch, 2012). Increased p21 expression is associated with a cell cycle arrest in either G1 or G2/M phase (Karimian et al., 2016). For largazole, low concentrations have been described to induce a G1 arrest, while higher doses are associated with a G2/M arrest (Liu et al., 2010). Previous studies have hypothesized that the G1 arrest is due to transcriptional activation, while strong transcriptional repression at higher doses induces a G2 arrest (Sanchez et al., 2018). This might explain that both a G1 arrest in TNBC cell lines and a G2/M arrest in the UIVC-IDC-4 culture are observed. A p21 induction has also been described in TNBC after Musashi targeting in breast, endometrial, and ovarian cancer and a similar phenotype, S phase decrease and G1 increase, was found (Troschel et al., 2020; Löblein et al., 2021; Götte et al., 2011).

This suggests that largazole is influencing the cell cycle similar to the MSI knockdown approaches, leading to a reduced proliferation and reduced migrating properties, which was consequently observed via DHM.

Moreover, DHM measurements demonstrated reduced migratory properties which is in line with attenuated invasive capacities as previously described for a combination of largazole and glucocorticoids (Law et al., 2013).

### 4.2 Largazole-associated gene expression changes

Largazole was previously described as an inhibitor of the Musashi RNA-binding proteins based on a docking analysis (Wang et al., 2020). However, the docking analysis was performed using the prodrug largazole, not considering protein-assisted hydrolysis resulting in the HDAC-active species, largazole thiol, which inevitably occurs in human cells or plasma. While largazole thiol may have other unidentified direct protein targets beyond HDACs, perhaps with lower affinity, the molecular and phenotypic effects are observed at nanomolar concentrations where class I HDAC activity is compromised, suggesting causality and not requiring direct Musashi inhibition. This is also supported by the fact that the same group also found that Musashi mRNA levels attenuated after largazole treatment which seems inconsistent with a functional protein inhibition.

Literature research indicated that HDAC inhibition leads to an increase of miR-125b levels, likely via a modulation of acetylated histone H3 (Wang et al., 2013). Additionally, miR-125b has been shown to decrease Musashi-1 expression via a luciferase-confirmed binding to the 3′UTR (Forouzanfar et al., 2021). While Musashi-2 was not specifically investigated in this study, we considered a similar mechanism highly likely given strong homology between both proteins and mRNAs. Our data confirmed this hypothesis as we found miR-125b-5p induced after largazole treatment and Musashi-1 and Musashi-2 levels repressed after miR-125b-5p overexpression. Additionally, we confirmed the effect of largazole on Musashi-1 and Musashi-2 levels in a time-dependent manner in all tested cell lines and cultures (Figures 5C–E). Finally, we found that largazole regulated Musashi protein levels in a miR-125b-5p-dependent manner: Anti-miR-125b-5p abrogated largazole-induced downregulation of Musashi expression, confirming our hypothesis of a largazole/miR-125b-5p/Musashi axis.

We consequently aimed to assess largazole-mediated changes on the binding partners of the Musashi RNA binding proteins. Numb, a repressor of the NOTCH pathway, was reported to be upregulated in Musashi knockdown approaches in TNBC (Troschel et al., 2020). However, we observed a downregulation of Numb in all tested cell lines and primary cell culture. Intriguingly, we nonetheless found NOTCH-3 largely downregulated, suggesting that Numb downregulation did not increase NOTCH activity. This is similar to a study on a Musashi inhibitor Ro 08–2750 (Brücksken et al., 2025). The reasons for the unexpected Numb downregulation remain unclear, but may involve an insufficient compensatory mechanism due to a previously described direct NOTCH downregulation following HDAC inhibition (Wang et al., 2018; Ferrante et al., 2020). HDAC inhibition itself was re-confirmed in our study for largazole. However, given numerous prior studies on the largazole-HDAC interplay (Al-Awadhi et al., 2020; Benelkebir et al., 2011), we did not further analyze this well-known relationship.

We observed a reduction of CD44 expression (Brücksken et al., 2025) as well as less spheroid formation (Haiduk et al., 2023; Löblein et al., 2021), similar to the Musashi knockdown approach. These findings point to a reduction in cancer stemness of our TNBC cell lines with CD44 as a known and ubiquitously expressed cancer stem cell marker associated with cancer initiation and aggressiveness (Guo et al., 2022). Conversely, CD24 was expressed at extremely low levels in all cultures. The observed reduction in mammosphere formation and size underlines a loss of stemness. We report both size and number of mammospheres, as is custom in the literature: Grimshaw et al. described a correlation between mammosphere size and tumorigenicity in metastatic breast cancer cells (Grimshaw et al., 2008), and this combined method is often used to analyze the prevalence of cancer stem cells as seen in a variety of similar studies (Grimshaw et al., 2008; Pece et al., 2010; Choi et al., 2017; Klopp et al., 2010). Notably, while mammosphere size is likely also governed by proliferative signaling, the ability to form spheres in a specific low-nutrient stem cell medium also depends on stem cell signaling.

For a broader understanding, RNA sequencing was performed. Key dysregulated hallmarks included “genome instability” (relevant for radioresistance and underlined by our findings in colony formation with irradiation and 53BP1 and yH2AX analysis (Figure 7) as well as in GO-BP analyses where an extensive number DNA repair-associated terms was found to be dysregulated), “resisting cell death” (relevant for cytotoxicity), and “tissue invasion and metastasis” (relevant for cell migration as demonstrated in our motility analysis via digital holographic microscopy (Figure 3)). All three hallmarks were also dysregulated after use of the Musashi inhibitor Ro 08-2750 (Brücksken et al., 2025), though other hallmarks not found here were also identified in that analysis.

### 4.3 Radiation-related effects

Our GSEA analysis underlined that DNA repair and cell cycle progression were key downregulated processes in TNBC after largazole application, supported by our findings in cell cycle analysis (Figure 2). Consequently, largazole itself also leads to more DNA double strand breaks in the TNBC cell cultures, most prominently after genotoxic treatment in the form of irradiation. These findings are new but in line with the general assumption that HDACs are involved in DNA damage repair mechanisms in cancer (Rajendran et al., 2011). Others have used DNA double strand break analyses to show that some HDAC inhibitors may attenuate early (Johnson et al., 2015) or late (Rivera et al., 2017) DNA double strand break repair. As we observe differences at both timepoints, our data suggests that DNA repair is consistently compromised.

Both early (El-Awady et al., 2003) and late (Noda, 2018) levels of post-irradiation DNA double strand breaks have been described as predictive of cellular survival. In accordance with these findings, we identified largazole as a radiosensitizer, similar to other HDAC inhibitors with studies in prostate cancer (Chinnaiyan et al., 2005) glioma (Chinnaiyan et al., 2005), and melanoma (Munshi et al., 2005), among others. Importantly, after initially finding little influence of largazole on the metabolically active cell population in non-malignant cells, we also identified no additional radiosensitizing effects in both keratinocytes and non-malignant breast epithelial cells. Intriguingly, MCF10A were substantially more resistant to radiotherapy instead, which is in line with studies in other low-proliferating non-malignant cells such as fibroblasts (Johnson et al., 2021). While we did not perform mechanistic analyses in non-malignant cells, these functional results reinforce that largazole disproportionately negatively affects cancer cells. This is in agreement with previous findings that healthy tissue is known to have a low pre-treatment expression of the Musashi proteins (Haiduk et al., 2023) and a low HDAC activity (Cui et al., 2018), both of which are targeted by largazole.

Our study offers intriguing insights into the anti-oncogenic properties of largazole, but some important questions remain open for further research. First and most importantly, further *in vivo* study in solid tumors seems necessary to solidify the understanding of the promising potential shown here. While our toxicity assay and radiation resistance analyses in non-malignant tissue suggest a vastly better tolerance for largazole application in non-cancerous tissue, additional experiments may help elucidate mechanistic changes induced by largazole in these cells, especially with regards to the increased radiation resistance seen in MCF10A cells. In this setting, a TNBC *in vivo* model should validate our findings. Second, while we identify a mechanism linking largazole to Musashi downregulation, additional connecting pathways are likely to exist and still need investigation, especially as many effects seen in our Western blot analyses were limited in size. Our mechanism also implies that other HDAC inhibitors may downregulate Musashi protein levels as well, a hypothesis that remains open for further research. Third, our study was not designed to clearly differentiate Musashi-associated and HDAC-associated effects. However, given the interplay we suggest, it seems likely that both dysregulations are closely intertwined after HDAC inhibitor use. Finally, the duration of largazole-associated effects has not been assessed and washout studies may be needed. Our data suggest that spheroid formation is reduced long-term despite previously published rapid metabolization of largazole within hours (Yu et al., 2014).

In summary, largazole influences cell growth, migration and cancer stem cell characteristics. Mechanistically, we found that largazole downregulated Musashi-1 and Musashi-2 levels via the miR-125b-5p. Gene expression data suggested that largazole targets cell cycle and DNA repair-associated pathways. Consequently, radiation-induced DNA double strand breaks were increased after largazole treatment, leading to its identification as a radiosensitizer.

## Data Availability

The RNA-Seq data presented in the study are deposited in the Gene Expression Omnibus (GEO) repository, accession number GSE305290.
