# Supplementary material for: Largazole targets Musashi protein expression via miR-125b-5p and sensitizes triple-negative breast cancer cells to radiation
Source: Front Pharmacol. 2026 Jun 23;17:1745079. doi: 10.3389/fphar.2026.1745079 (PMC13337822; doi:10.3389/fphar.2026.1745079)
Supplement: Supplementary file 1 [file Supplementaryfile1.docx]

Supplementary Material 1


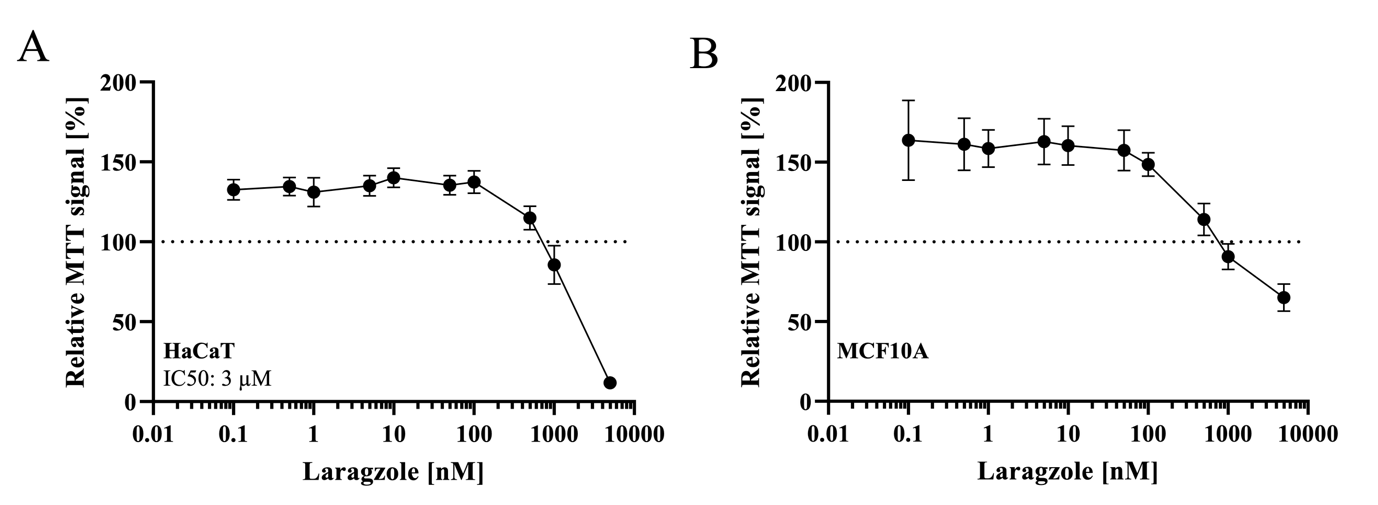


Figure 1: Cytotoxic effects of largazole in HaCaT and MCF10A. The cell lines HaCaT and MCF10A were treated with different concentrations of largazole. Treatment tolerance differed, as evidenced by different IC50 values by MTT assay: (A) IC50 value was 3 µM in HaCaT cells and no IC50 value could be calculated for MCF10A (B). All experiments were repeated at least three times in independent replicates. Error bars indicate standard deviation.


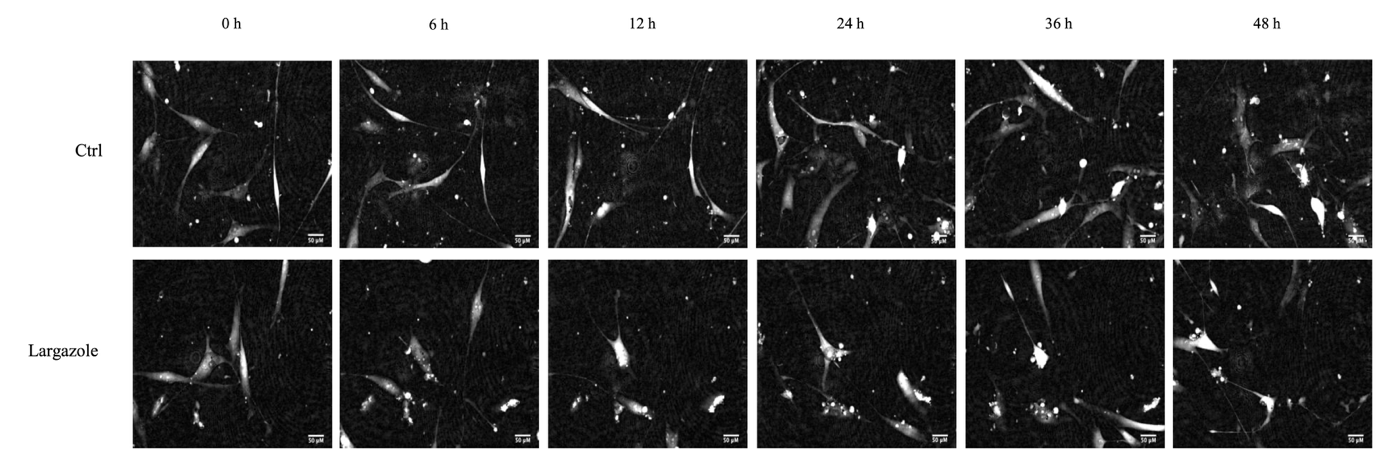


Figure 2: UIVC-IDC-4 primary cells +/- largazole treatment in digital holographic microscopy measurements. Images are presented from several timepoints during longitudinal measurements for control (Ctrl) and largazole-treated samples.


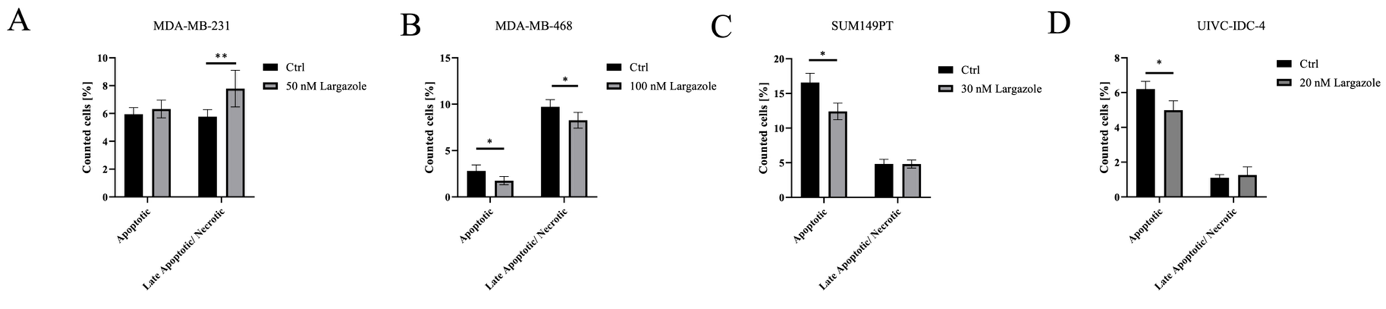


Figure 3: Apoptosis is slightly reduced upon largazole treatment in some TNBC cultures. The TNBC cell lines MDA-MB-231 (A), MDA-MB-468 (B), SUM149PT (C) as well as the primary cell culture UIVC-IDC-4 (D) were treated with largazole, stained and measured via flow cytometer using the Annexin V Apoptosis Detection Kit I (BD Biosciences). All experiments were repeated at least three times in independent replicates. Error bars indicate standard deviation.


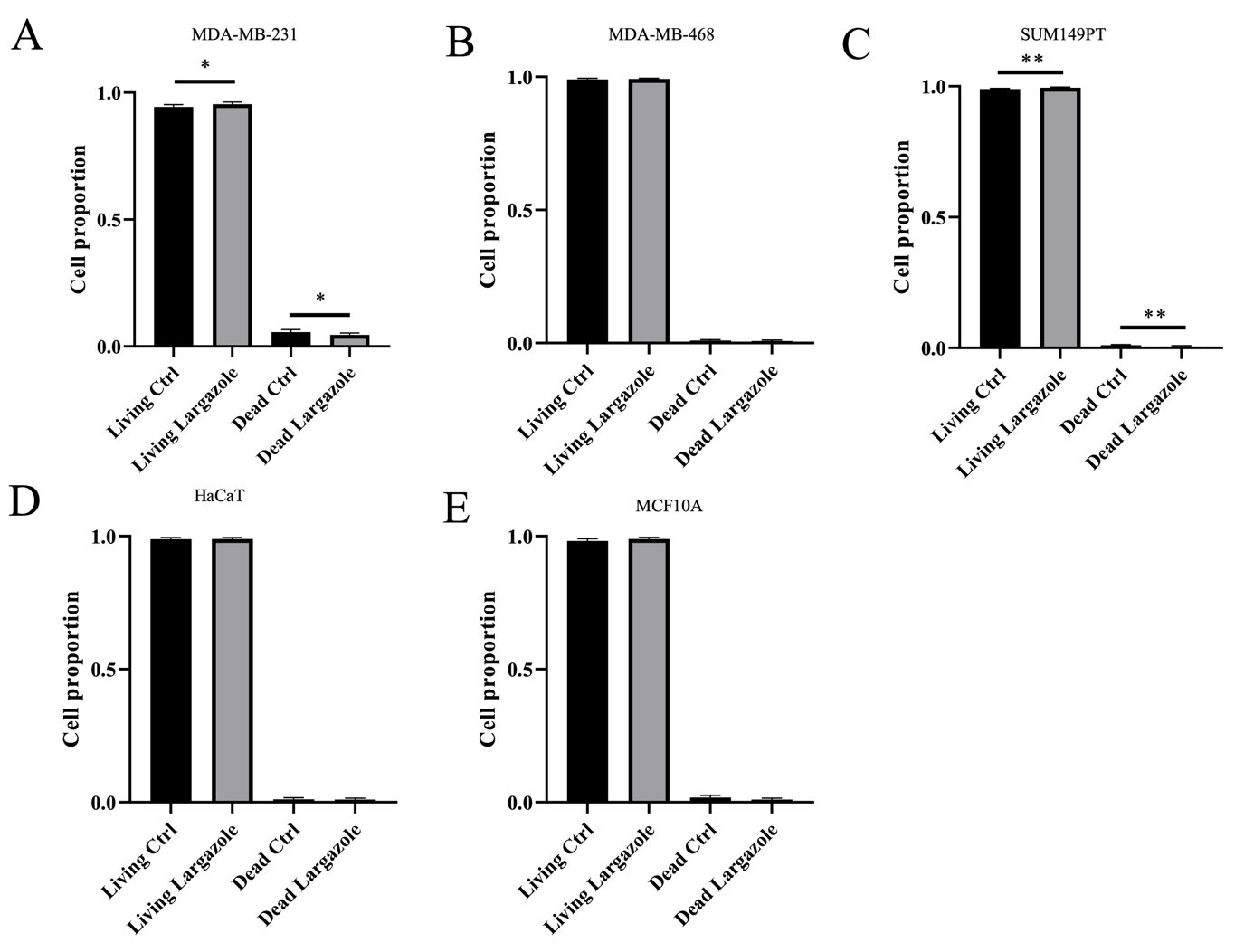


Figure 4: Largazole has no impact on cell death. The TNBC cell lines MDA-MB-231 (A), MDA-MB-468 (B), SUM149PT (C), HaCaT (D), and MCF10A (E) were treated with largazole, harvested and stained with trypan blue for identification of living or dead cells. All experiments were repeated at least three times in independent replicates. Error bars indicate standard deviation.
