# Supplementary figures and images for: Largazole targets Musashi protein expression via miR-125b-5p and sensitizes triple-negative breast cancer cells to radiation
Source: Front Pharmacol. 2026 Jun 23;17:1745079. doi: 10.3389/fphar.2026.1745079 (PMC13337822; doi:10.3389/fphar.2026.1745079)

## Slide 1
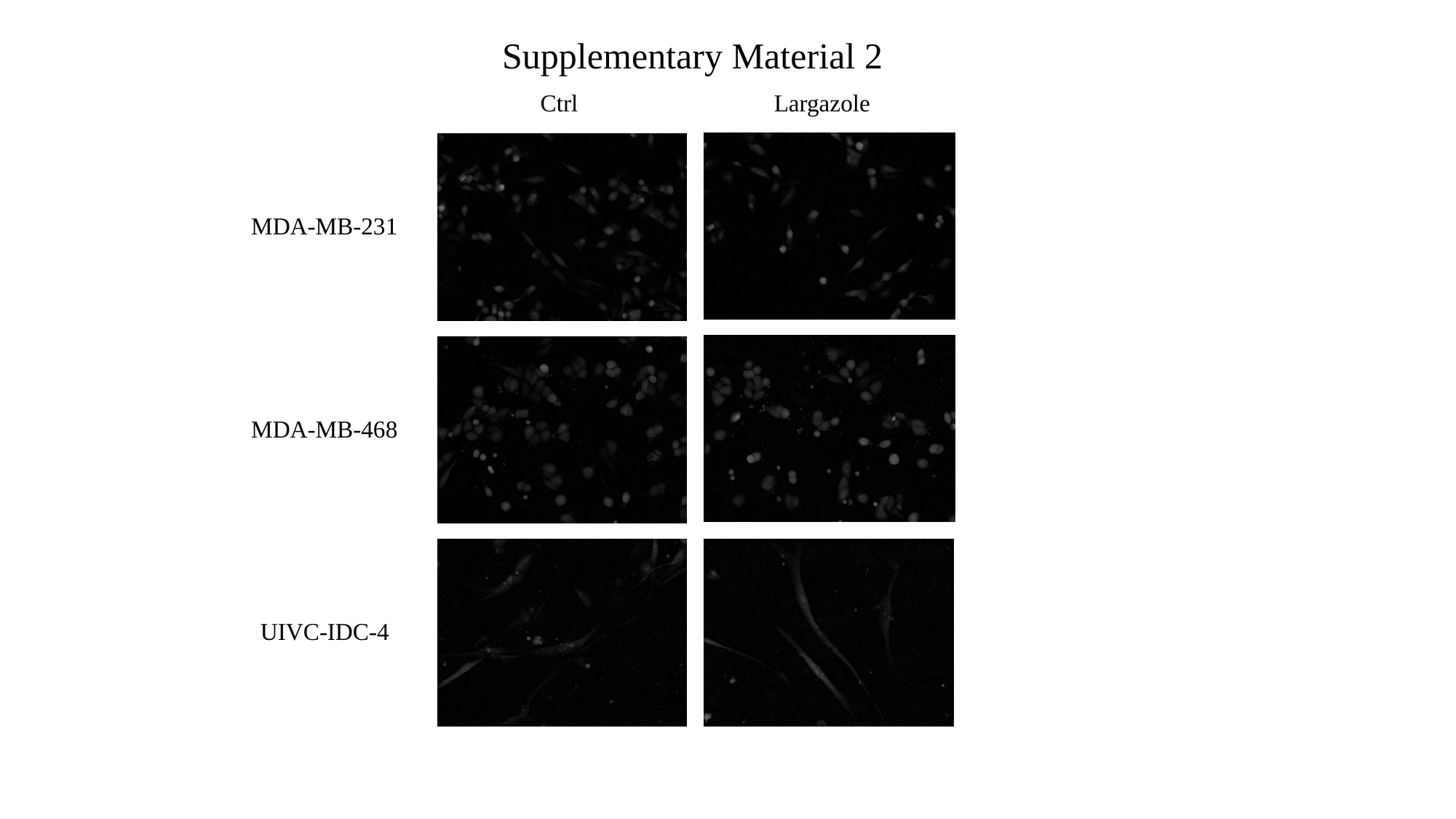

Supplementary Material 2
Ctrl
Largazole
MDA-MB-231
MDA-MB-468
UIVC-IDC-4

Supplement: Supplementary file 2 [file Supplementaryfile2.pptx]
